# Supplementary material for: Content-rich biological network constructed by mining PubMed abstracts
Source: BMC Bioinformatics. 2004 Oct 8;5:147. doi: 10.1186/1471-2105-5-147 (PMC528731; doi:10.1186/1471-2105-5-147)
Supplement: Additional File 5 — The original Chilibot query results of the term "long-term potentiation (LTP)" and 22 other terms, limiting the latest references analyzed to the years 1990, 1995, 2000, and 2004. [file 1471-2105-5-147-S5.bz2 › chilibotAdditionalFile5/ltp2000/html/LTP_ERK.html]

 


 **LTP** and **ERK** 
  
Found 11 abstracts in PubMed,  **11 abstracts were retrieved and analyzed**.  


---

 Search Google  |
 PDF files only 
|  EDU domain only 

---

**Interactive relationship** (e.g. stimulation, inhibition, etc)

- **ERK**  plays a regulatory role in induction of  **LTP**  by theta frequency stimulation and its modulation by beta adrenergic receptors.  Ref: 10595521 Neuron, 1999
- We also show that inhibition of the MAPK  **ERK**  cascade by the MAPK kinase MEK inhibitor SL327 prevented CREB and Elk 1 phosphorylation, and  **LTP**  dependent gene induction, resulting in rapidly decaying  **LTP** .  Ref: 10844026 J Neurosci, 2000
- We found that  **ERK**  was activated in area CA1 after the induction of both 200 Hz  **LTP**  and TEA  **LTP** .  Ref: 10777769 J Neurosci, 2000
- Activation of extracellular signal regulated kinase  **ERK**  has been shown to be necessary for NMDA receptor dependent long term potentiation  [ **LTP** ]   **LTP** .  Ref: 10777769 J Neurosci, 2000
- **ERK**  is required for the early phase of  **LTP**  elicited by brief 5 Hz stimulation, as well as for  **LTP**  elicited by more prolonged 5 Hz stimulation when paired with beta1 adrenergic receptor activation.  Ref: 10595521 Neuron, 1999
- Here we show that induction of long term potentiation  [ **LTP** ]   **LTP**  in the dentate gyrus in vivo leads to rapid phosphorylation and nuclear translocation of MAPK  **ERK** .  Ref: 10844026 J Neurosci, 2000
- We have found that  **ERK**  also regulates hippocampal neuronal excitability during 5 Hz stimulation and thereby regulates forms of long term potentiation  [ **LTP** ]   **LTP**  that do not require macromolecular synthesis.  Ref: 10595521 Neuron, 1999
- Overall, our observations indicate that activation of the  **ERK**  signaling cascade is necessary for NMDA receptor independent  **LTP**  in area CA1 but not in area CA3 and suggest a divergence in the signaling cascades underlying NMDA receptor independent  **LTP**  in these hippocampal subregions.  Ref: 10777769 J Neurosci, 2000
- By contrast,  **ERK**  plays no role in  **LTP**  elicited by a single 1 s 100 Hz train.  Ref: 10595521 Neuron, 1999
- Moreover,  **ERK**  mediated changes in excitability are selectively required for some forms of  **LTP**  but not others.  Ref: 10595521 Neuron, 1999
- Inhibition of the  **ERK**  signaling cascade with either PD 098059 or U0126 prevented the induction of both 200 Hz  **LTP**  and TEA  **LTP**  in area CA1.  Ref: 10777769 J Neurosci, 2000

**Parallel relationship** (e.g. studied together, co-existance, homology, etc.)

- Furthermore, the full expression of the late phase of long term potentiation  [ **LTP** ]  L  **LTP**  and L  **LTP**  associated CRE mediated transcription requires  **ERK**  activation, suggesting that the activation of CREB by  **ERK**  plays a critical role in the formation of long lasting neuronal plasticity.  Ref: 9808472 Neuron, 1998
- We have studied the effects of interleukin 6 IL 6 on the expression of paired pulse facilitation PPF, posttetanic potentiation PTP, and long term potentiation  [ **LTP** ]   **LTP**  in the CA1 region of the hippocampus as well as on the activation of the signal transducer and activator of transcription 3 STAT3, the mitogen activated protein kinase  **ERK**  MAPK  **ERK** , and the stress activated protein kinase c Jun NH 2 terminal kinase SAPK JNK.  Ref: 10899938 J Neurochem, 2000
- Because activation of the extracellular signal regulated kinase  **ERK**  has been shown to be necessary for long term potentiation  [ **LTP** ] , we investigated the regulation of  **ERK**  in long term depression LTD in the adult hippocampus in vivo.  Ref: 10617120 J Neurochem, 2000
- The IL 6 induced inhibition of PTP and  **LTP**  was accompanied by a simulation of STAT3 tyrosine phosphorylation and an inhibition of MAPK  **ERK**  dual phosphorylation, in the absence of changes in the state of activation of SAPK JNK.  Ref: 10899938 J Neurochem, 2000
- Given the possible role of altered gene expression in the late phase of  **LTP**  and in long term memory, we evaluated the capacity of the MAPK  **ERK**  extracellular signal regulated kinase to regulate phosphorylation of the transcription factor cAMP response element binding protein CREB in hippocampal area CA1.  Ref: 11016081 Acta Neurobiol Exp (Wars), 2000
- For example, the mitogen activated protein kinase, or extracellular signal regulated kinase  **ERK** , signaling cascade has increasingly been shown to be important for the induction of gene expression and long term potentiation  [ **LTP** ] .  Ref: 10652372 J Biol Chem, 2000
- The MAPK  **ERK**  cascade targets both Elk 1 and cAMP response element binding protein to control long term potentiation  [ **LTP** ]  dependent gene expression in the dentate gyrus in vivo.  Ref: 10844026 J Neurosci, 2000
- Thus, MAPK  **ERK**  activation is required for  **LTP**  dependent transcriptional regulation and we suggest this is regulated by two parallel signaling pathways, the MAPK  **ERK**  Elk 1 pathway targeting SRE and the MAPK  **ERK**  CREB pathway targeting CRE.  Ref: 10844026 J Neurosci, 2000
- We studied the role of  **ERK**  in three forms of NMDA receptor independent  **LTP**   **LTP**  induced by very high frequency stimulation 200 Hz  **LTP** ,  **LTP**  induced by the K channel blocker tetraethylammonium TEA TEA  **LTP** , and mossy fiber MF  **LTP**  MF  **LTP** .  Ref: 10777769 J Neurosci, 2000
- Analysis of phosphorylation of the two tyrosine kinase substrates trkA and  **ERK**  in synaptosomes prepared from untetanized and tetanized dentate gyrus revealed that  **LTP**  was associated with increased phosphorylation of both proteins.  Ref: 10560922 Hippocampus, 1999
